# When Livestock Genomes Meet Third-Generation Sequencing Technology: From Opportunities to Applications

**DOI:** 10.3390/genes15020245

**Published:** 2024-02-15

**Authors:** Xinyue Liu, Junyuan Zheng, Jialan Ding, Jiaxin Wu, Fuyuan Zuo, Gongwei Zhang

**Affiliations:** 1 College of Animal Science and Technology, Southwest University, Rongchang, Chongqing 402460, China; luckyliuxy@163.com (X.L.); 13896120486@163.com (J.Z.); djl20212023@163.com (J.D.); wjiaxin0225@163.com (J.W.); zfuyuan@163.com (F.Z.); 2 Beef Cattle Engineering and Technology Research Center of Chongqing, Southwest University, Rongchang, Chongqing 402460, China

**Keywords:** third-generation sequencing, gene assembly, transcriptome, epigenetics

## Abstract

Third-generation sequencing technology has found widespread application in the genomic, transcriptomic, and epigenetic research of both human and livestock genetics. This technology offers significant advantages in the sequencing of complex genomic regions, the identification of intricate structural variations, and the production of high-quality genomes. Its attributes, including long sequencing reads, obviation of PCR amplification, and direct determination of DNA/RNA, contribute to its efficacy. This review presents a comprehensive overview of third-generation sequencing technologies, exemplified by single-molecule real-time sequencing (SMRT) and Oxford Nanopore Technology (ONT). Emphasizing the research advancements in livestock genomics, the review delves into genome assembly, structural variation detection, transcriptome sequencing, and epigenetic investigations enabled by third-generation sequencing. A comprehensive analysis is conducted on the application and potential challenges of third-generation sequencing technology for genome detection in livestock. Beyond providing valuable insights into genome structure analysis and the identification of rare genes in livestock, the review ventures into an exploration of the genetic mechanisms underpinning exemplary traits. This review not only contributes to our understanding of the genomic landscape in livestock but also provides fresh perspectives for the advancement of research in this domain.

## 1. Introduction

The utilization of high-throughput sequencing technologies in molecular genetics research has become increasingly prevalent. Next-generation sequencing (NGS) technology, represented by the Illumina sequencing platform, is known for its advantages in terms of accuracy and low cost. However, the short read lengths result in the inability to sequence certain repetitive genomic sequences, leading to assembly errors and gaps in the genome assembly. Additionally, it lacks the capability for direct DNA/RNA sequencing, rendering it unable to fully meet the evolving technological demands of modern biology. Third-generation sequencing (TGS) technologies, exemplified by single-molecule real-time (SMRT) sequencing from Pacific Biosciences (PacBio) and nanopore sequencing from Oxford Nanopore Technologies (ONT), have emerged as the leading methods in the genomic, transcriptomic, and epigenetic field due to their significant advantages, such as long read lengths, real-time base sequencing, direct sequencing, and shorter processing times [1,2]. SMRT utilizes two sequencing modes, continuous long reads (CLR) and circular consensus sequencing (CCS), displaying outstanding advantages in the sequencing of complex gene structures, the identification of SNP variations, the detection of gene structures, such as full-length transcriptomes, alternative splicing (AS) and fusion genes, and the discrimination between monozygotic twins [3,4]. ONT technology, driven by motor proteins, directly threads DNA/RNA strands through nanopores, representing a direct, real-time sequencing approach that eliminates the need for PCR amplification. This approach preserves base modification information and enables accurate quantitative analysis, significantly improving the accuracy, read length, and throughput of ONT sequencing [5]. TGS has addressed the shortcomings of NGS in genomic composition and transcript isoform analysis, establishing itself as the preferred technology for gene function studies [6].

## 2. Advances of TGS in the Genomic Research of Livestock

**Figure 1:**
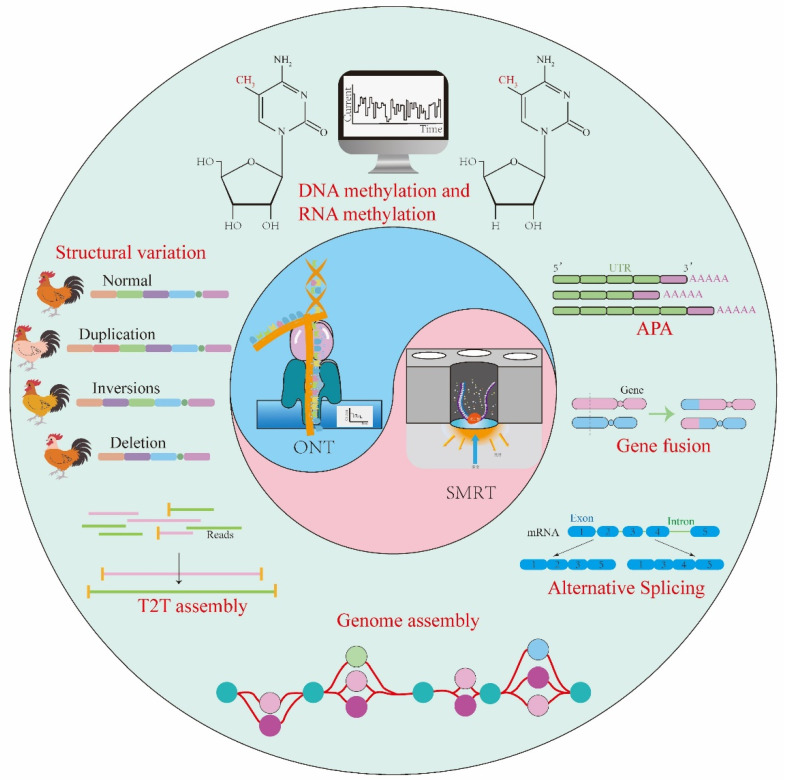
The application of TGS in the genomic research on livestock, including genome assembly, structural variation detection, transcriptome sequencing, and epigenetic analysis.

The TGS technology has found extensive applications in the genomics and molecular genetics research of livestock, serving as a powerful tool for the deciphering of genome structure, functionality, and evolution. Firstly, it efficiently conducts comprehensive sequencing of the livestock genome, providing a holistic genomic dataset that facilitates the discovery of genetic variations and the elucidation of gene functions [7]. Secondly, through long-read sequencing, TGS can more accurately detect structural variations within the genome, including insertions, deletions, and inversions, thereby offering more precise genomic information for the study of relevant traits [8]. Moreover, this technology proves advantageous in the superior assembly of complex genomes, particularly those in livestock characterized by highly repetitive sequences and chromosomal structures with intricate features [9]. Simultaneously, in epigenetic studies, the long reads generated by TGS enable a more comprehensive and accurate resolution of DNA methylation and RNA modification patterns [10], shedding light on the intricate network governing gene expression regulation in livestock. Finally, the application of TGS in genomic selection involves the comprehensive and accurate analysis of large-scale individual genome sequencing data, facilitating genomic selection strategies that accelerate genetic improvement in livestock breeds and enhance breeding efficiency [11]. This paper primarily summarizes the research progress of TGS technologies in livestock genetics, specifically in genome assembly, structural variation detection, transcriptome sequencing, and epigenetic analysis (Figure 1), providing novel insights for further genetic studies in livestock.

### 2.1. Progress in Genome Assembly Using TGS in Livestock

A complete genome is a prerequisite for the obtaining of accurate genetic information, the precise exploration of genetic details, and the deciphering of the mechanisms of genetic variation. It provides a theoretical foundation for the in-depth analysis of genetic features in germplasm resources and promotes the development of genomics and molecular breeding. Genome assembly involves the assembly of sequenced fragments into a complete genomic sequence; it uses two primary methods: de novo assembly and mapping-based assembly. Additionally, the assembly algorithms include overlap–layout–consensus (OLC) and the de Bruijn graph (DBG) [12]. Following the advent of NGS, the de novo sequencing of livestock species became feasible, typically by constructing a reference genome based on a representative breed [13]. TGS technologies demonstrate remarkable advantages and enormous potential in genome assembly research, and they are widely applied in various animals, such as cattle [14,15], gayals [16], yaks [17,18], buffalos [19,20], sheep [21,22], goats [23,24], pigs [25,26], chickens [27,28], ducks [29], and geese [30] (Table 1).

### 2.2. Progress in Pan-Genome Using TGS in Livestock

**Figure 2:**
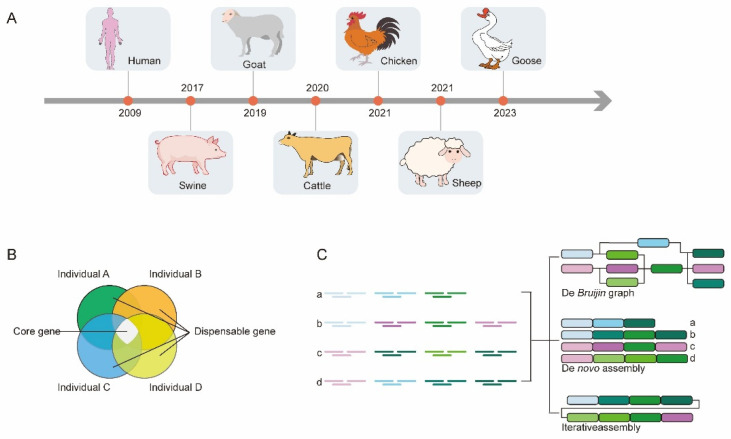
The development process and construction methods of pan-genomic research. (**A**) Numerous species have developed pan-genomes, including emiliania huxleyi. (**B**) Pan-genomes comprise core genes, dispensable genes, and strain-specific genes. (**C**) Pan-genome construction strategies include iterative assembly, de novo assembly, and graphical pan-genomes.

Due to the limited genetic diversity covered by a single reference genome, the concept of the pan-genome was introduced by researchers [31,32]. The pan-genome encompasses the sum of all genomic information within a species, capturing more genetic diversity. Presently, numerous species have developed pan-genomes based on NGS data, including the human [33], pig [34,35,36], goat [37], cattle [38,39], chicken [40], sheep [41] and goose species [30] (Figure 2A). The pan-genome comprises core genes, dispensable genes, and strain-specific genes [42]. Core genes are shared by all individuals of a species and are generally associated with biological functions and major phenotypic features. Dispensable genes exist in some but not all individuals, reflecting species-specific adaptations or unique biological traits [43]. Strain-specific genes are unique to specific individuals, indicating individual-specific traits (Figure 2B). The pan-genomic analyses conducted on these species have yielded novel insights. The pan-genome construction strategies include iterative assembly, de novo assembly, and graphical pan-genomes [44] (Figure 2C). These strategies utilize large-scale sequencing data, deep sequencing of a small number of individuals, and graph-based data structures to represent the gene sequences and structures of species.

### 2.3. Progress in Telomere-to-Telomere Assembly Using TGS

Telomeres are specialized terminal regions of linear chromosomes in eukaryotes; they play crucial roles in cell division and chromosome replication. The need to understand the characteristics of repetitive sequences within genes for better genetic mechanism studies has made telomere assembly a research hotspot. With the rapid development of sequencing technologies, a new era of “Telomere-to-Telomere (T2T) assembly” is unfolding [45]. Through T2T assembly, the intricate structures and functions of complex regions in the genome, such as telomeres and centromeres, can be deeply explored. Achieving gapless chromosome assembly from telomere to telomere holds significant importance for the in-depth exploration of the complex region of centromeres in the genome and further uncovers crucial genetic variations [46]. Researchers have combined TGS with genome assembly technologies to successfully accomplish T2T assembly in various species, including humans [47,48] and chickens [28], obtaining high-quality genomes. Huang et al. pioneered the completion of a full-genome map for domestic chickens, using Huxu breed chickens as material and identifying the six missing chromosomes in the original genome assembly. The complete chicken chromosome models are useful in the reconstruction of the karyotype of the vertebrate ancestor [28].

**Table 1:** TGS techniques for genome assembly and pan-genome research in livestock.

Species	Feature	Breed	Sequencing Platform	Key Findings	Publication Year	Reference
*Bos taurus*	Cattle	1.OⅹO F1 2.NⅹB F1 3.GⅹP F	ONT	Constructed haplotype-resolved genomes for cattle and related species, established a pan-genome for cattle, and quantified structural diversity	2022	[14]
*B. taurus*	Cattle	Southern Yellow Cattle	PacBio-SMRT	Confirmed genetic diversity in the southern yellow cattle population in China, identified gene introgression events from five different wild cattle species	2023	[15]
*B. taurus*	Cattle	Hainan Cattle, Mongolian Cattle	ONT	Discovered significant structural variations influencing environmental adaptability in Chinese yellow cattle	2023	[49]
*Bos frontalis*	Cattle	Gayal	PacBio-SMRT	Conducted chromosome-level genome assembly for Dulong cattle	2023	[16]
*Bos grunniens*	Yak	Yak	ONT	Obtained high-quality chromosome-level genomes for wild and domestic yaks, a structural variation catalog for yaks, and a single-cell transcriptome atlas of lung tissues	2022	[17]
*B. grunniens*	Yak	White Yak	ONT	Revealed genetic introgression of unique structural variations in the color-sided yellow cattle, resulting in the creation of the color-sided Yak. Subsequent genetic variations gave rise to the white Yak	2023	[18]
*Bubalus bubalis*	Buffalo	Water Buffalo	PacBio-SMRT	Generated a detailed genomic map for water buffalo (2n = 50) and performed chromosome-level genome assembly	2019	[19]
*B. bubalis*	Buffalo	Swamp-type Water Buffalo, River-type Water Buffalo	PacBio-SMRT	Attained high-quality chromosome-level reference genomes for swamp-type water buffalo (2n = 48) and river-type water buffalo (2n = 50)	2020	[20]
*Ovis aries*	Sheep	Dorper Sheep	ONT	Revealed the genetic basis of allele-specific expression (ASE) genes and specific phenotypic traits in Dorper sheep	2022	[21]
*O. aries*	Sheep	15different breeds of sheep	PacBio-SMRT	Constructed high-quality pan-genome maps for different sheep breeds	2023	[22]
*Capra hircus*	Goat	Saanen Dairy Goat	PacBio-SMRT	Assembled the reference genome Saanen_v1 for Saanen dairy goats	2021	[23]
*C. hircus*	Goat	Tibetan Goat	PacBio-SMRT	Unveiled PAPSS2 as a key gene not only for high-altitude adaptation in goats but also a significant gene in genetic introgression analysis	2022	[24]
*Sus scrofa*	Pig	Tibetan Pig, Jinhua Pig, and 8 other breeds	ONT	Completed pan-genome maps for Anqing Liubai Pig, Laiwu Pig, Meishan Pig, Min Pig, Rongchang Pig, Wuzhishan Pig, Yorkshire Pig, European Wild Boar, etc.	2023	[25]
*S. scrofa*	Pig	Duroc	PacBio-SMRT	Assembled the reference genome Sscrofa11.1 for pigs from scratch	2020	[26]
*Gallus gallus*	Chicken	Huxu Chicken	ONT	First published complete genome atlas (T2T) for vertebrates; characterized the epigenetics of the W chromosome; elucidated the origin, sequence structure, and diversity of chicken centromeres	2023	[28]
*G. gallus*	Chicken	Chickens from Four Continents	PacBio-SMRT	Established the pan-genome of chickens, identified new coding genes, long non-coding RNAs, and new gene families; identified new gene clusters for studying collinearity	2022	[50]
*G. gallus*	Chicken	Wenshang Lu Hua Chicken	PacBio-SMRT	Obtained a high-quality chromosome-level reference genome for the Wenshang Lu Hua chicken	2023	[51]
*Anas platyrhynchos*	Duck	Peking Duck, Shaoxing Duck, and Mallard	PacBio-SMRT	Assembled chromosome-level high-quality genomes for Peking Duck, Shaoxing Duck, and mallard, refuting the “missing gene hypothesis” in birds	2021	[29]

O: Original Braunvieh cattle (Bos taurus taurus); N: Nellore (Bos taurus indicus); G: gaur (Bos gaurus) bull; B: Brown Swiss (Bos taurus taurus) cattle; P: Piedmontese (Bos taurus taurus) cow.

### 2.4. Understanding the Genetic Mechanisms of Livestock Traits Using TGS

The emergence of TGS technology has had a profound impact on the field of life sciences. Compared with traditional NGS, multiple aspects of TGS technology have shown significant advantages. The long read feature of TGS enables it to better cope with gene regions with complex structural variations (SVs) and repetitive sequences, particularly when exploring the genetic mechanism of animal traits; thus, it can more accurately reveal the genetic basis of animal traits. In addition, TGS technology has higher sequencing accuracy, which helps to more accurately identify mutation sites and mutations in the genome that may be related to animal traits and to provide strong support for in-depth research on the genetic mechanism of animal traits. Furthermore, TGS technology has improved sequencing speed, saved time costs, and enabled more efficient association analysis of large samples and genomic association research. This helps in the discovery of more genes and variation sites related to animal traits. In summary, TGS technology has significant advantages in the exploration of the genetic mechanisms of animal traits, providing a powerful tool for a deeper understanding of the genetic basis of animals.

SV is an important part of the pan-genome, including insertion, deletion, duplication, translocation, inversion, and so on. SVs are significant contributors to genetic variations in livestock [52]. TGS technologies can detect structural variations such as deletions, duplications, inversions, translocations, and more (greater than 50bp) [53]. In the detection of SV events, researchers have found that long-read sequencing is more convenient than short-read sequencing [54]. The state-of-the-art SV callers, i.e., cuteSV [55], NanoSV [56], NanoVar [57], Sniffles [58], SVIM [59], and PBSV (https://github.com/PacificBiosciences/pbsv (accessed on 9 February 2024)) can be used for TGS data.

Copy number variation (CNV) is a crucial component of SV; it describes the molecular phenomenon of genomic sequence repetition and plays a key role in promoting population diversity and both micro- and macroevolutionary processes in humans and animals [60]. Magini et al. found that nanopore sequencing, when compared with the current most advanced CNV detection techniques, can reduce the time required for CNV detection at the same resolution, and ONT has greater stability in identifying chimeric CNVs [61]. In this review, we focused on the application of TGS in livestock.

#### 2.4.1. Understanding the Genetic Mechanisms of Traits in Ruminants

In cattle, researchers constructed a graphical pan-genome for 10 southern Chinese yellow cattle breeds, utilizing the high accuracy and continuity advantages of local directional haplotype genomes. They clarified five gene infiltration events from different wild cattle species in the population history of southern Chinese yellow cattle [15]. Liu et al. built a graphical pan-genome for yak and cattle species, discovering that nearly 90% of the domestic yak genome contains genes that have infiltrated from yellow cattle. They identified that the cause of the white yak coat color is due to the infiltration of an SV spanning the *KIT* gene [18]. Genetic infiltration from unique SVs in color-sided yellow cattle led to the formation of color-sided yaks; subsequently, the infiltrated structural variation produced a new genetic variation which resulted in the production of white yaks [18]. Meanwhile, Gao et al. constructed high-quality chromosome-level reference genomes for wild yaks and domestic yaks. Using a combination with a single-cell map of lung tissue, they indicated that the development of lung endothelial cells and their function in low-oxygen adaptation may be influenced by SV [17]. Concurrently, Xia et al. assembled high-quality chromosome-level genomes for Hainan cattle and Mongolian cattle. Utilizing various omics technologies, they identified significant SVs influencing the environmental adaptability of Chinese yellow cattle [15]. Researchers using the nanopore platform, analyzed alleles related to horns and found that the size of the inserted sequences at the Celtic locus ranged from 181 to 206 bp. Through alignment with ARS-UCD1.2, repetitive sequences were displayed on the Celtic locus, providing direct evidence for the presence of the Celtic locus in the Australian Brahman cattle population [62].

In sheep, Li et al. assembled high-quality genomes for 15 sheep breeds and constructed high-quality pan-genomic maps for different sheep breeds. They identified numerous divergent allele genes, complex multi-allele variations, and crucial candidate mutation sites related to tail length and thickness traits in sheep [22]. In goats, Li et al. constructed a goat pan-genome web interface for data visualization by comparing nine de novo assemblies from seven sibling species of domestic goats with ARS1 and by using resequencing and transcriptome data from goats for verification [37]. The researchers revealed the strongest high-altitude adaptation signature in Tibetan goats at the PAPSS2 locus by using data from 331 genomes and 104 transcriptomes, which provided evidence that interspecific introgression contributed to the high-altitude adaptability. This study expanded the gene repertoire of hypoxia adaptation in highland-dwelling mammals and provided new insights into their evolutionary origins [24].

#### 2.4.2. Understanding the Genetic Mechanisms of Traits in Monogastric Animals

NGS technology has been used in the pan-genome assembly and gene structural variation detection in pigs; 12 de novo pig assemblies from Eurasia were compared to identify the missing sequences from the reference genome, and 72.5 Mb of non-redundant sequences (~3% of the genome) were found to be absent from the reference genome [36]. Furthermore, Li et al. systematically analyzed the presence/absence variation (PAV) of the coding sequences in 250 sequenced individuals from 32 pig breeds in Eurasia by constructing a pan-genome [35]. Jiang et al. utilized 11 pig breeds to construct a graphical pan-genome for pigs, revealing 206 Mb of new sequences and detecting 183,352 non-redundant structural variations. They explored the significant role of structural variations in high-altitude adaptation in Tibetan pigs [25].

In horses, Viluma et al. used PacBio sequencing technology to sequence bacterial artificial chromosome (BAC) clones spanning the major histocompatibility complex class II (MHC class II) of horses for the first time. They discovered numerous CNV sites, providing important resources for the association study of immune-mediated diseases in horses and the evolutionary analysis of genetic diversity in this region [63].

In chickens, 664 sample data were used to construct a pan-genome of chickens in order to gain a deeper understanding of the changes in the genome structure during evolution. *IGF2BP1*, as the causal variant of the chicken body size quantitative trait locus located at chromosome 27, was found for the first time. Therefore, the chicken pan-genome is a useful resource for biological discovery and breeding [40]. However, researchers performed a de novo assembly on 20 chicken individuals worldwide, constructing the first high-quality pan-genome based on de novo assembly for birds. They identified new coding genes, long non-coding RNAs, and novel gene clusters [50]. The genome sequences of a reference chicken genome (GRCg7b) and the resequencing reads of 15 Silkie chickens (8 males and 7 females) onto the Silkie genome were aligned to identify a total of 9,337,467 SNPs and 920,864 small insertions and deletions (indels, referring ≤50 bp) [27]. Using two different methods, the PanGenome Graph Builder (PGGB) and Minigraph-Cactus, pangenome references of the chicken genome were constructed. This new genome reference paradigm will better identify the mutations responsible for specific phenotypes, provide tools for in-depth research on the chicken genome structure and variation, and help in the understanding of chicken genetic characteristics and evolutionary processes; in the cultivation of chickens, it is necessary that new sustainability and disease resistance capabilities are met [64]. By performing nanopore sequencing on different chicken strains, they discovered that the high local variation rate of SV and the negative selection of harmful SV events drive the rapid evolution of piRNA [65].

In ducks, Zhu et al. assembled chromosome-level high-quality genomes for Peking ducks, Shaoxing ducks, and mallards, annotated thousands of new protein-coding genes, and refuted the hypothesis of missing genes in birds by confirming the presence of presumed “missing genes” in the genome [29]. The researchers conducted pan-genome estimations for gene PAV detection construction analysis by comparing various genome versions (Tianfu goose, Sichuan white goose, and Zhedong white goose) to investigate genomic sequences beyond the single-reference genome sequence. Although this strategy proved valuable in obtaining 612 Mb of a new sequence, 2,813 new genes, and a total of 20,503 genes across the pan-genome, the limitations, such as shorter contig lengths in the assembly of contigs using second-generation sequencing data, were not to be neglected [30].

## 3. Application of TGS in the Transcriptome of Livestock

Long-read RNA-sequencing technologies have now reached a mature stage, having already been used to study transcript structures, novel transcripts, and APA, as well as for early allele-specific analyses [4,66]. Applying TGS technology to sequence RNA from different tissues allows the analysis of alternative splicing, the identification of new transcripts (genes), and the optimization of gene structures, which reveal the hidden transcriptional complexity in humans [67] and in livestock [68,69]. The expression product of a gene is a protein, and alternative splicing may lead to protein changes, influencing biological phenotypes. Therefore, the study of alternative splicing is a focal point in current molecular genetic research in livestock [70]. The analysis of the TGS results for alternative splicing identification and regulatory network analysis across pigs and chickens has revealed that alternative splicing affects various biological processes [71,72]. Alternative splicing generates different transcript isoforms, allowing a single gene to be transcribed into multiple transcripts, which can be translated into different protein subtypes to regulate different phenotypes or exert distinct physiological functions [73,74].

### 3.1. Application of TGS in the Transcriptome of Ruminant Animals

In recent years, alternative splicing has been widely applied in ruminant animals, including cattle, sheep, and so on. Alternative splicing plays a crucial role in the transcriptional changes induced by environmental disturbances. Researchers sequenced RNA from different periods of bovine fat cells using ONT sequencing. The direct sequencing of full-length RNA accurately reflected the RNA modification status, detecting modifications at the single-base level and their potential roles in gene expression and selective splicing in fat cells, enhancing our understanding of the mechanisms underlying fat formation in cattle [75]. Additionally, studies on different mRNA modifications implied that TGS technology plays a crucial role in the discovery of new transcripts in animals (Table 2). In cattle, researchers analyzed the full-length transcriptome using both SMRT and ONT technologies. Based on the full-length transcriptome, numerous alternative splicing events, alternative polyadenylation (APA) sites, novel isoforms, novel lncRNAs, and transcription factors provided a more comprehensive foundation for the exploration of the diversity of the cattle transcriptome. The full-length transcriptome was refined, revealing differentially expressed transcripts among various tissues [69,76]. Yuan et al. used SMRT technology to perform RNA long-read sequence analysis on muscle tissues from male sheep of different meat qualities and hybrid strains. They annotated the sheep genome and discovered a new isoform, ANKRD23, which is associated with tenderness and is potentially regulated by the CCCTC-binding factor (CTCF) [77].

### 3.2. Application of TGS in the Transcriptome of Monogastric Animals

Meanwhile, many studies have focused on the application of the transcriptome in monogastric animals such as pigs, chickens, and birds. In pigs, researchers sequenced the *Longissimus dorsi* muscle in pigs from different intramuscular fat using ONT sequencing, discovering new splicing bodies related to skeletal muscle development and fatty acid metabolism [71]. In poultry, Guan et al. sequenced different tissues of White Leghorn chickens using ONT sequencing, revealing tissue-specific transcripts. Two brain tissues (cerebellum and cortex) showed the highest number of expressed transcripts and sites, while the reproductive tissues (testes and ovaries) exhibited the most tissue-specific transcripts [78]. Genes related to follicle development were discovered through the analysis of the full-length transcriptome obtained from avian sequencing [72,79,80,81].

**Table 2:** Studies of TGS technology in the transcriptome.

Species	Feature	Breed	Sequencing Platform	Key Findings	Publication Year	References
*B. taurus*	Cattle	Hereford Cattle	ONT	Discovered tissue-specific transcripts in cattle, with the testes exhibiting the most complex transcriptome	2021	[69]
*B. taurus*	Cattle	Simmental Cattle	PacBio-SMRT	Analyzed the full-length transcriptome of Simmental cattle, providing a foundation for refining the cattle draft genome annotation, optimizing genome structure, and comprehensively characterizing the cattle transcriptome	2021	[76]
*O. aries*	Sheep	(Dorper × Hu) × Hu sheep; Dorper × (Dorper × Hu sheep)	PacBio-SMRT	Revealed the transcriptome complexity and identified many candidate transcripts in tail fat, which could enhance the understanding of molecular mechanisms behind tail fat deposition	2021	[82]
*C. hircus*	Goat	Chinese Cashmere Goat	PacBio-SMRT	Showed the superiority of full-length transcriptome data in gene annotation; more such data are required to improve the gene annotation for goat genome and that of other species	2023	[83]
*S. scrofa*	Pig	Large White Pig × Min Pig F2 Generation	ONT	Discovered differentially expressed mRNA isoforms involved in skeletal muscle development and fatty acid metabolism	2022	[71]
*G. gallus*	Chicken	White Leghorn Chicken	ONT	Identified the most tissue-specific transcripts in reproductive tissues (testes and ovaries) of chickens	2022	[78]
*G. gallus*	Chicken	Hy-Line Brown Chicken	ONT	Revealed mRNA and lncRNA expression differences between pre-GCs and post-GCs during chicken follicle selection; discovered significant estrogen-induced expression of three *DHCR7* isoforms	2023	[72,79]
*Cairina moschata*	Duck	Muscovy Duck	ONT	Obtained the full-length transcriptome of Muscovy duck follicles, providing structural and functional annotations for new transcripts	2021 2022	[80,81]

## 4. Advances of TGS in Epigenetic Studies of Livestock

### 4.1. Application of TGS in DNA Methylation Modification

DNA methylation is a common epigenetic modification found in prokaryotic and eukaryotic genomes. It plays a crucial role in the regulation of gene expression. Methylation in the gene promoter regions and at the transcription start sites can inhibit gene transcription, thereby exerting a significant impact on the regulation of biological activities [84].

Currently, the detection of epigenetic modifications can be achieved through both SMRT and ONT sequencing [85,86]. SMRT sequencing data often exhibit relatively weak signals for epigenetic modifications, requiring high coverage at specific sites to determine the presence of epigenetic gene modifications. This method is primarily applied in the analysis of smaller genomes, such as in single-cell epigenetic sequencing and the corresponding modification of the high-throughput detection of 5-methylcytosine (m5C) in bacterial genomes [87,88]. The duration of the fluorescence signal and the interval between two signals generated by SMRT correspond to the kinetics of DNA synthesis. The duration of the fluorescence signal at DNA methylation site 5-mC is much longer than that at non-methylation site C. Differences in duration and interval times allow the detection of various types of epigenetic modifications. The characteristics of SMRT sequencing, such as long read lengths and insensitivity to GC repeats, enable the complete detection of CpG island sites in high-GC repeat regions in epigenetics [89]. In the context of cloned cattle systems, the application of TGS to the study of differentially methylated regions (DMRs) revealed an increase in DNA methylation in multiple genes, including PEG1-DRM [90]. The application of nanopore sequencing in DNA methylation detection provides a new avenue for epigenetic research. Currently, ONT is used to evaluate the epigenetic characteristics of human cell line DNA, particularly CpG methylation and chromatin accessibility. CpG methylation and chromatin accessibility on long-stranded DNA are simultaneously evaluated by applying exogenous labeling of open chromatin with GpC methyltransferase [91]. In addition, ONT can also detect the copy number changes in circulating tumor DNA (ctDNA) and cancer-specific methylation changes in tumor patients [92].

TGS technology has significant advantages in gene methylation detection. Its long read length property enables researchers to detect DNA methylation more accurately; thus, the mystery of the epigenome is revealed more comprehensively. In livestock, ONT is expected to provide strong support for genetic improvement and disease prevention and control. Through the methylation detection of the animal genome, researchers can have a deep understanding of the genetic basis of animal growth and development, reproductive performance, disease resistance, and other traits and can provide key information for breeding work. In addition, ONT has important value in disease diagnosis and prevention and control, which helps in the development of more effective disease diagnosis methods and prevention and control strategies and to reduce the economic losses of animal husbandry. With further research and technological advancements, the study of DNA methylation is expected to make significant progress in the field of epigenetics.

### 4.2. Application of TGS in RNA Epigenetic Modifications

In recent years, research in epi-transcriptomics has provided a new direction for animal studies. The relationship between RNA epi-transcriptome and animal phenotypes and traits is of significant importance for understanding the essence of life and revealing evolutionary processes. Epigenetic studies are based on the structure and modifications of RNA molecules, including N6-methyladenosine (m6A), 5-methylcytosine (m5C), and the spatial structure of RNA. Currently, 150 types of RNA modifications have been discovered in various fields [93]. Epigenetics is closely related to the growth and development of animals, with methylation being one of the rich modifications in epigenetics. m6A is the most common methylation modification in mRNA; it dynamically and reversibly regulates various life activities, including gene expression, RNA metabolism, and protein translation [94]. The study of RNA epigenetic modifications relies on the advancements in sequencing technologies. ONT technology allows direct sequencing of RNA through the monitoring of the changes in the present caused by the passage of individual molecules through a membrane-embedded nanopore. This method enables the direct detection of RNA base modification sites, producing long reads that cover the entire transcript, making it a promising alternative for studying m6A [95].

While ONT sequencing technology has been widely applied in the study of RNA epigenetic modifications in plants and microorganisms, its application in livestock is relatively limited. However, the role of m6A epigenetics in livestock is substantial. Qin Jiang et al. found that m6A methylation plays a crucial regulatory role in the uniform deposition of fat and muscle in Jin Hua pigs and is associated with the decomposition metabolism of Landrace pigs. They further showed that MTCH2 promotes fat generation in muscles in an m6A-dependent manner [96]. m6A RNA methylation, through the regulation of metabolites by gut microbiota, such as folic acid and butyric acid, affects the nutrition, absorption, and metabolic mechanisms of livestock [97]. ONT sequencing technology is expected to provide targets for precise nutrition and targeted regulation in different livestock.

## 5. Prospects

With the rapid development of molecular biology technologies, TGS has gained widespread application in the research of superior breeding and genetic reproduction in livestock due to its advantages such as long reads, real-time base sequencing, and short turnaround time. The advantages of the low cost, high yield, and high accuracy of NGS technology have made it widely used in large-scale sequencing. However, compared with TGS technology, the short read length of NGS increases the difficulty and error rate of gene assembly. At the same time, the PCR technology used in NGS increases the error rate of sequencing. The long read length of TGS technology helps reduce the splicing cost in bioinformatics, providing convenience in subsequent data analysis and interpretation [98]. The use of PacBio Iso-Seq can detect transcripts that would have otherwise been missed by RNA-seq [99]. Further research found that the utility of Iso-Seq can uncover hidden mammalian transcriptional complexity, not seen by RNA-seq alone, which examined the correlation between RNA-seq and Iso-Seq estimations of relative transcript abundance and their predictions of differential gene expression [68]. However, several considerations need attention in the application of TGS: (1) Error Rates and Sequencing Artifacts: The issue of base error rates and sequencing artifacts remains a challenge in TGS applications. Researchers are addressing this by improving reagent purity, developing rapid detection kits, and enhancing the accuracy of original read lengths. The development of more efficient and precise sequencing methods and error correction modules provides possibilities for the addressing of these challenges [100,101]. (2) Nanopore Technology for Single-Molecule Sequencing: The accuracy, read length, and throughput of nanopore technology for sequencing single long DNA and RNA molecules have significantly improved. This calls for the development of new experimental techniques and bioinformatics methods to fully exploit nanopore long-read sequencing in the study of genomes, transcriptomes, epigenomes, and transcription [102]. (3) Expanding Applications in Livestock Genomics: While TGS has found broad applications in the genomic and transcriptomic analysis and sequencing of livestock, its exploration in the epigenetics of livestock breeding is relatively limited and not comprehensive. Therefore, future efforts should focus on leveraging TGS for epigenetic studies in livestock and explore the interactions among epigenetic modifications. This represents a promising direction for future research in livestock breeding [102].

In summary, TGS technology has been extensively applied in various domains related to livestock genomics, including genome assembly, detection of structural variations, transcriptome sequencing, and epigenetic analysis. As the costs of TGS continue to be controlled and sequencing functionalities are further optimized, it is anticipated that it will become a routine technology in livestock breeding research. Its role is expected to be pivotal in discovering rare genes, cultivating superior individuals, conducting population-based genetic breeding, and performing single-cell whole-transcriptome sequencing.
